# Uptake of three doses of HPV vaccine by primary school girls in Eldoret, Kenya; a prospective cohort study in a malaria endemic setting

**DOI:** 10.1186/s12885-018-4382-x

**Published:** 2018-05-11

**Authors:** Hillary Mabeya, Sonia Menon, Steven Weyers, Violet Naanyu, Emily Mwaliko, Elijah Kirop, Omenge Orango, Heleen Vermandere, Davy Vanden Broeck

**Affiliations:** 1 Moi University, Eldoret, Kenya; 2 International Center of Reproductive Health, Ghent University, De Pintelaan 185 P3, 9000 Ghent, Belgium; 3 CDC Foundation, Atlanta, Georgia USA; 4 Ghent University, Ghent, Belgium

**Keywords:** Kenya, Gardasil, HPV vaccination, Determinants, Parents/guardians, Malaria

## Abstract

**Background:**

All women are potentially at risk of developing cervical cancer at some point in their life, yet it is avoidable cause of death among women in Sub- Saharan Africa with a world incidence of 530,000 every year. It is the 4th commonest cancer affecting women worldwide with over 260,000 deaths reported in 2012. Low resource settings account for over 75% of the global cervical cancer burden. Uptake of HPV vaccination is limited in the developing world. WHO recommended that 2 doses of HPV vaccine could be given to young girls, based on studies in developed countries. However in Africa high rates of infections like malaria and worms can affect immune responses to vaccines, therefore three doses may still be necessary. The aim of this study was to identify barriers and facilitators associated with uptake of HPV vaccine.

**Methods:**

A cross-sectional survey was conducted at Eldoret, Kenya involving 3000 girls aged 9 to 14 years from 40 schools. Parents/guardians gave consent through a questionnaire.

**Results:**

Of all 3083 the school girls 93.8% had received childhood vaccines and 63.8% had a second HPV dose, and 39.1% had a third dose. Administration of second dose and HPV knowledge were both strong predictors of completion of the third dose. Distance to the hospital was a statistically significant risk factor for non-completion (P: 0.01).

**Conclusions:**

Distance to vaccination centers requires a more innovative vaccine-delivery strategy and education of parents/guardians on cervical screening to increase attainment of the HPV vaccination.

## Background

All women are potentially at risk of developing cervical cancer at some point in their life, yet it is avoidable cause of death among women in Sub- Saharan Africa with a world incidence of 530,000 every year. It is the 4th commonest cancer affecting women worldwide with over 260,000 deaths reported in 2012. Low resource settings account for over 75% of the global cervical cancer burden [1].

Cervical cancer remains the most common cancer in Eastern and Central Africa with an estimated with estimated age standardized rates (ASRs) of 42.7 and 30.6 respectively compared with 2–5 in Western Europe and Australia. Mortality is highest in Eastern Africa at 27 per 10,000 compared to less than 2 per 100,000 in Western Asia/Europe, Australia and New Zealand [1].

Among the Eastern African countries, Kenya has a high cervical cancer burden which is the leading cause of cancer deaths in women of reproductive age and the 2nd most frequent cancer among women in Kenya. The incidence of cervical cancer cases in Kenya is estimated at close to 2500 with annual deaths close to just over 1500 with an average life lost estimated at 25 years. The incidence is projected to double by the year 2025 if no efforts are put to mitigate the increment. Secondary prevention for women in reproductive age is only 3% compared with 40–70% in high resource countries [2–6].

HPV vaccination as a primary prevention method is supplemented by screening later in life. This is because vaccines do not protect against all high-risk and potential high-risk HPV types. Vaccines to prevent cervical cancers are now available. The quadrivalent (Gardasil 4) vaccine protecting against human papilloma virus (HPV) types 16/18/6/11 and the bivalent (Cervirax) vaccine protecting against infection with HPV-16/18 were both originally used as a three dose schedule. A third vaccine, the nonavalent HPV (Gardasil 9) vaccine protecting against HPV types 16/18/6/11/31/33/45/52/58 has been approved by Food and Drug Administration (FDA) in 2014 in the USA [7]. The cost and logistical difficulties and cost in resource-constraint settings has delayed or slowed implementation of the original three-dose vaccine regimen. GAVI Alliance supported HPV vaccination in 2011 and if less than three dose schedule is adopted in these countries, there will be an increase the implementation of HPV vaccination programs in resource-constraint countries [4, 7]. Three vaccines are now being marketed in many countries as primary prevention intervention tool. HPV 16 and 18 are responsible for approximately 70% of cervical cancer cases globally. Gardasil and Nonavalent also prevent anogenital warts that are caused by HPV 6 and 11 [8].

World Health Organisation has recommended offering HPV vaccination to girls at ages 9–13 and Kenya has developed a comprehensive strategy to address cervical cancer, which includes rollout of HPV vaccination among girls, with the purpose of vaccinating girls naïve to the targeted types. This roll out of HPV vaccine among girls naïve to the targeted types, is akin to the one which took place in Rwanda, Uganda, Cameroon, Tanzania, Lesotho and South Africa, which have introduced HPV vaccine to prevent cervical cancer through school-based programme [5–7, 9, 10, 11].

WHO has recommended bivalent or quadrivalent vaccine in the 2-dose schedule with a 6-month interval between doses for females less than 15 years old, including females 15 years or older at the time of the second dose based on available studies, before the females become sexually active. A third dose is recommended at least 6 months after the first dose, if the interval between the two doses is shorter than 5 months and for 15 years old or more who are known to be immunocompromised and/or HIV-infected or are already sexually active. This 2 dose recommendation will lead to cost saving and other logistic problems of delivering the vaccine and compliance [2, 7].

Education and knowledge by health personnel and parents/guardians about cervical cancer, HPV and HPV vaccines is necessary for vaccine uptake and hence enhanced primary prevention of cervical cancer [12–15].An HPV immunization program must also consider the unique challenges that HPV vaccination implementation faces, including a recommended schedule consisting of 2 doses for 9 to 14 year old girls and 3 doses for those above 15 years and if they are immunocompromised. So far, there is no published research from Kenya on risk factors for partial HPV regimen administration.

This study sought to explore barriers and facilitators that undermine or promote adherence to 3 doses HPV vaccine in a malaria and helminthic endemic western Kenya and explore policy implications for HPV vaccine introduction. Watson-Jones et al. in their attitudes and access to HPV vaccination among Maasai pastoralists and slum dwellers study found challenges to an HPV vaccination programme included early age of sex and marriage, lack of parental support, school absenteeism and drop-out, population mobility and distance from services. Despite little prior knowledge of cervical cancer and HPV, and school-based vaccination, communities were interested in receiving HPV vaccination. Adequate social mobilization supplemented by out-reach activities, were considered important facilitating factors to achieve high coverage [16].

HPV vaccines in populations whose immunological system may be challenged by multiple co-infections such as malaria and helminth infections are needed measure long term protection and advice on booster doses. Studies have shown that malaria and helminth infections have an effect on long term vaccine responses and hence more research is needed [17, 18].

In 2007 GAP reached more than 445,000 girls in 21 countries and several have implemented a national vaccination program. The burden of cervical cancer remains a big problem in developing world and the GAP initiative through the vaccine delivery will continue to play a valuable role as countries consider large-scale implementation. The demonstration programs and comprehensive analysis of the vaccine delivery has been published in BMC Public Health in May 2012 [19, 20].

In sub-Saharan Africa, many countries are considering the implementation of HPV vaccination. These countries have a high prevalence of endemic malaria and helminth infections that can act as immunological modulators and impact responses to standard immunizations. These infections can impact immune responses to vaccinations [21–27].

Sub-Saharan Africa except South Africa has high incidences of malaria. In Kenya, malaria remains a major cause of morbidity and mortality with more than 70% of the population at risk of the disease (MOH 2014). More than four million cases of malaria are reported annually in Kenya. A 5.1% mortality ratehas been reported among patients admitted with severe malaria. The malaria burden in Kenya is not homogeneous. Western Kenya, areas around Lake Victoria and on the cost present the highest risk [28–30].

A high HPV immunogenicity regardless of the presence of malaria and helminth infections among young girls and women in Tanzania study was found. There was some evidence of enhanced antibody titres to HPV vaccine genotypes in participants with malaria parasitaemia. Additional research on the impact of parasitic infection on the long-term duration of protection from HPV vaccines is warranted [31].

There is a paucity of information on the association between malaria and/or helminthic disease and cervical disease progression. The way malaria and helminths affect the immune response to HPV vaccine long term protection needs to be investigated. Studies should explore differences between levels of HPV 16/18 antibodies after administration of the vaccine among girls exposed to malaria/and or helminthic infections [32].

## Methods

### Promotion

Through the GARDASIL Access Program (GAP), the Moi Referral and Teaching Hospital (MTRH) was granted 9600 doses of the quadrivalent vaccine to administer to young girls in Eldoret, Kenya. MTRH is Kenya’s second largest referral hospital with a bed capacity of 550 of which 100 are in reproductive health wards, with a service population of 13 million people. It is located in Eldoret town about 325 km from Nairobi in Western part of Kenya. It is a teaching hospital for both undergraduate and post graduate students from Moi University.

Eldoret is a principal city in western Kenya. It also serves as the capital of Uasin Gishu County. Lying south of the Cherangani Hills, the local elevation varies from about 2100–2700 (7000–9000 ft). The population was 289,380 in the 2009 census and it is currently the fastest growing town in Kenya.

Eldoret experiences highland malaria transmission if temperatures become warm and rainfall encourages mosquito breeding. Residents of Eldoret do not develop immunity and suffer much more serious illness than their lowland neighbours.

Promotion of the program was restricted to a number of randomly selected government primary schools, although other girls from the community were not refused if appeared at the vaccination site. A hospital-based vaccination was chosen to reduce the costs of the program.

The vaccines were offered to girls of age 9 to 14 years old, from ten primary schools in Eldoret Municipality. These schools were randomly selected until a total of about 4000 eligible girls was reached, expecting a coverage of around 75% (3000/4000). Permission to roll out the vaccination was given from the County Director of Education who in turn instructed the head teachers of involved schools. A training of trainers (TOT) process was set in motion, with teachers first being trained by health staff and through the provision of leaflets the teachers were asked to instruct students and parents/guardians on HPV vaccination and cervical cancer.

Vaccination took place from May 2012 to March 2013. After a signed consent was obtained from the parent/guardian, nurses vaccinated the girls. A vaccination card with a next appointment was given upon the first and second dose. Additionally, caregivers who failed to show up on the schedule day were given a reminder by nurses about the second and third dose. Due to a low response during the first 3 months of the program, other schools in the County, government and private, were also invited to participate from August 2012 onwards through posters in the hospital and radio. In September 2012, the program stopped administering the first dose after reaching 3000 girls with a view to guaranteeing sufficient vaccines for the remaining doses.

### The survey

This was done before administration of the vaccine. The questionnaire consisted of two parts. The first part collected information on sociodemographic characteristics, including religion and age of school girls. The second part consisted of questions regarding the guardian’s knowledge of HPV, Pap smear knowledge and whether the female guardian/spouse had undergone a Pap smear test. The survey used in this study was designed by public health specialists and physicians. Data was collected from 22/04/2012 to 23/04/2014 by data collection team. The survey was pilot-tested by for comprehensibility before study commencement.

Interviewers administered questionnaires in English, and verbal interpretation in Swahili based on the participant’s request.

### Data analysis

Following data checking and cleaning, analysis was undertaken using STATA version 12. Corporation, College Station, TX, USA. In order to assess whether a median or mean should be given for continuous variables, a Shapiro-Wilk test at an alpha level of 5% was performed to assess whether the variable was parametric. We checked to see whether the missing data (< 5%) was missing completely at Random by performing the Little’s MCAR test. Prob> chi-square = 0.5472 so indicating that the data missing is completely at random and is unlikely to have altered our results and multiple imputation is not required.

Dates for second dose, third dose, Pap smear undertaken, HPV knowledge was dichotomized into the two categories, yes and no. Google maps were used to calculate distance from School to vaccination center. Distance to health centre in kilometres and age were both used as a continuous variable and the non-parametric Mann Whitney rank sum test was performed as it could not be assumed that the continuous variables were normally distributed. Bivariate logistic regression was used to measure the strength of the association, with the dependent variable being the number of dose. Statistical analyses were performed using ‘svy’command to account for clustering in making variance estimates, with school being the primary sampling unit.

A statistical test was considered significant if the *p*-value is 0.05 and borderline significant it was between 0.06 and 0.1.

## Results

### Population characteristics

The median age range of schoolgirls whose caregivers were interviewed was 12 (IQR: 10–13), of which, 2853 (99.2%) was Christian and 22 (0.8%) Muslim. 843 (28.1%) had a male parent, 2154 (71.8%) had a female parent and 3 (0.1%) had both. 786 (27%) of parents/guardian had no knowledge of Pap tests. 307(14.4%) of all female guardians underwent a Pap test; 824 (27.2%) of parents/guardians had knowledge of HPV. Of all the school girls eligible for this study 2808(93.8%) had childhood vaccines and 1933(63.8%) had second HPV dose, and 1182 (39.1%) had a third dose. The mean time elapsed between the second HPV vaccine and the first vaccine was 32.5 days (SD 22.7), the third HPV vaccine and the second vaccine 148 days (SD: 17.5). The median time elapsed between the first HPV vaccine dose and the third HPV vaccine dose was 175 days (IQR: 168–182) (see Table 1).

**Table 1:** Population characteristics

Population characteristics	Number	Percentage and Confidence Interval
Age	12 (IQR: 10–13)	
Religion		
Christian	2853/2875	99.20% (95%CI: 99.0–99.5%)
Muslim	22/ 2875	0.80% (95%CI .0.5–1.2)
Caregiver		
Female	2154/3000	71.8% (95%CI: 70.2–73.4)
Male	843/3000	28.1% (95% CI: 26.5–29.7)
Knowledge		
Knowledge of Pap Smear tests	786/2913	27% (95%CI: 25.4–28.6)
HPV knowledge among caregivers	824/2853	28.90% (95%CI:.27.2–30.6)
Female caregiver having had cytological screening	307/ 2126	14.40% (95%CI: 13.0–16.0)
Vaccines		
Childhood vaccines	2808/ 2994	93.80% (95%CI: 92.9–.94.6)
HPV second dose	1933/ 3026	63.80% (95%CI: 62.13–65.6)
HPV third dose	1182/ 3026	39.10% (95%CI: 37.3–40. 8)
Mean time between second HPV dose and first dose	33 days (SD 22.7)	
Mean time between third HPV dose and second dose	148 days (SD: 17.5)	
Median time elapsed between first and last HPV dose	175 days (IQR: 168–182)

**Table 2:** Barriers/facilitators of full HPV vaccine regime

Barriers/facilitators of full HPV vaccine regimen	Odds Ratio	95% CI	*P* value
Administration of second dose of HPV	61.1	40.9–91.0	< 0.0001
HPV knowledge	1.2	1.1–1.5	0.008
Male guardian	1.14	1.0–1.3	0.04
Pap Smear test knowledge	1.2	1.0–1.4	0.08
Childhood vaccine	1.2	0.8–1.6	0.4
Religion	1.1	0.4–2.5	0.9
Female guardian who had a Pap Smear test	0.9	0.7–1.2	0.6
Differences in medians			
Age			0.1
Distance			0.01

### Barriers and facilitators of HPV vaccine uptake

Administration of second dose of HPV and HPV knowledge were both strong predictors of taking the third dose of HPV vaccine (OR: 61.1; *p* <  0.001; 95% CI = 40.9–91.0) and (OR: 1.2;*p* = 0.008; 95%CI 1.1–1.5) respectively. A Mann Whitney test revealed that distance to health facility was a statistically significant barrier (p.0.01).

A 14% higher odds of administration of all three doses was found for girls who had a male guardian (*p* = 0.04; CI: 1.0–1.3). A marginal association was observed between Pap test knowledge of the guardian and third HPV vaccine (OR: 1.2; p:0.08; CI:1.0–1.4).

No statistically significant risk factors were observed for childhood vaccine (OR: 1.2; p: 0.4; CI: 0.8–1.6), religion (OR:1.1; p: 0.9; CI: 0.5–2.5) and female guardian who had a Pap test OR 0.9; p:0.6; CI:0.7–1.2. A Mann Whitney rank sum test did not reveal a significant difference in age medians between those who had the third HPV vaccine and those who did not. (p: 0.1). (See Table 2).

## Discussion

Three studies have been conducted in Kenya on actual HPV vaccination, our study and Ministry of Health GAVI HPV demonstration project in Kitui Kenya (2015). This research builds on limited research in Kenya on HPV vaccine acceptability for young girls and sought to explore factors associated with administration of the HPV vaccine, which is to our knowledge the first study to explore bottlenecks and facilitators for receiving 2 or 3 doses of HPV vaccine regimen. We have a vaccine uptake of 64% of two doses compared with other countries with less than 50%. Kenya is considering a national HPV vaccine program through GAVI after a successful pilot in 2015.

Our study revealed that only about 40% of school girls who had a first HPV dose got the third dose. Our low full uptake findings echo in Becker-Drep’s study et al. [15] on HPV vaccine acceptability among Kenya women, where women expressed a strong preference for a vaccine that would require only one dose as opposed to a vaccine requiring three doses [15]. In malaria endemic settings like Western Kenya, uptake of three doses may be negatively affected hence the need for more studies on the effect of malaria on long term vaccine protection and dosing.

Our very strong statistically significant association observed between administration of the second HPV dose and third HPV dose within the six-month time period that was initially stipulated by the WHO before the 2 dose regimen was introduced in 2014 suggests that the majority of guardians who are favourable to a second dose of HPV vaccine are willing and also understand the necessity of having their daughters receive a third HPV dose.

Vermandere et al. qualitative study on implementation of HPV vaccine program in Eldoret in Eldoret by key stakeholders showed that caretakers and teachers poorly understood cervical cancer and linked it with modern lifestyle and nonconforming sexual behavior. And few had ever heard about the vaccination opportunity and felt uncomfortable to discuss cervical cancer. Teachers needed support from health care workers to address questions from parents and there was distrust towards HPV vaccines [33].

Masika et al. [34] found out that knowledge about HPV vaccine among school teachers was low to moderate but high vaccine acceptability hence may affect uptake. Empowering teachers to be vaccine champions in their community may be a feasible way of disseminating information about HPV vaccine and cervical cancer [34].

Distance was found to be a significant predictor of HPV vaccine uptake, which suggests that a different vaccine delivery strategy be envisaged. This observation also concurs with Vermandere et al. (2012) observations that acceptability of the HPV vaccine may not translate in higher HPV uptake, lest operational problems are addressed for parents.

As expected since the outcomes of interest were facilitators and barriers for HPV protection after 1st dose and not uptake of the first dose, it was anticipated that no significant association would then be found between Christian and Muslim religion. With less than 1% of the study population reporting a religion other Christianity, we expected a slightly higher percentage given that the Muslim population in our study setting is close to 10% further studies should be conducted to find out any relationship between religion and vaccine acceptability and uptake.

Intuitively, we would have expected female parents/guardians to be significant predictor of three dose vaccine administration; however our results show that male parent/guardians were significantly associated with three dose regimen administration.

Also, whilst we observed a strong association between HPV knowledge and HPV vaccine uptake, unexpectedly, no statistically significant association was detected with undergoing Pap smear test by female parent/guardian, which may be indicative of the scarcity of information delivered to women receiving cytological screening on the necessity of HPV vaccine uptake for cervical cancer prevention.

### Strength and limitations

The study’s strength is that is our big sample size and that it is the first study to our knowledge that explores facilitators and barriers for HPV vaccine regimen uptake. However, our limitations include lack of data on potential known confounders, including meteorological conditions and time used to travel to the vaccine center as well as other potential unknown confounders which may not have been identified.

WHO guidelines 2014 and the CDC guidelines of 2016 have recommended 2 doses of HPV vaccine to girls less than 15 years old except those that are immunocompromised and HIV infected and 3 doses for those 15 years and older, immunocompromised, HIV infected and this may hamper generalizability of our findings [7].

#### Policy implications and research gaps

Our findings highlighting the low HPV vaccine uptake has a number of policy implications which could be addressed within the future Kenyan National Cervical Cancer Prevention strategic Plan (https://www.k4health.org/toolkits/Kenya-health-national-cervical-cancer-prevention-program-strategicplan-2012-2015. The observations derived from our study suggest that the plan could also promote screening for women so that they subsequently understand the importance of HPV vaccination and the completion of the recommended dose regimen. With research ongoing with respect to one dose HPV, implications on HIV, malaria/helminth infestation should be taken into consideration.

As distance to the clinic was found in our study to be a strong predictor of HPV vaccine uptake, a school-based vaccination, which has a track record of being more efficient [35], may be an alternative strategy providing that school attendance among girls reaches a certain threshold. Empowering teachers to be vaccine champions in their community may be a feasible way of disseminating information about HPV vaccine and cervical cancer and subsequently improve uptake.

Lastly, our current lack of knowledge of operational bottlenecks in Kenya for HPV vaccination uptake would benefit from qualitative studies to inform future quantitative survey tools. Qualitative studies are needed to illuminate our finding concerning why male guardians appear to be conducive to higher uptake of the HPV vaccine regimen.

## Conclusion

Findings from this study suggested a need to consider school-based vaccination and more active education of caregivers to increase attainment of HPV vaccination regimen. Promoting screening among women so that they, in turn be targeted by an awareness campaign on the importance of HPV vaccination for cervical cancer prevention.

Qualitative methods are urgently needed to illuminate our study’s quantitative findings and to inform future time series of cross-sectional surveys to reflect the trends of knowledge and barriers locally.

## Abbreviations

ASRs: Age Standardized Rates
CDC: Centers for Disease Control and Prevention
FDA: Food and Drug Administration
GAP: GARDASIL Access Program
GAVI: Global Alliance for Vaccines and Immunization
HIV: Human Immunodeficiency Syndrome
HPV: Human Papilloma Virus
TOT: Training of Trainers
USA: United States of America
WHO: World Health Organization
